# Anterior atlantoaxial motion preservation fixation technique for axis complex fractures (odontoid process with Hangman’s fractures) and technique notes

**DOI:** 10.1038/s41598-024-51367-2

**Published:** 2024-01-06

**Authors:** Qilin Lu, Jin Tang, Wei Xie, Xianzhong Mei, Hui Kang, Ximing Liu, Feng Xu, Xianhua Cai

**Affiliations:** 1 Department of Orthopedics, The Affiliated Hospital of Wuhan Sports University, Wuhan, Hubei People’s Republic of China; 2 Department of Orthopedics, Shenzhen Pingle Orthopedic Hospital, Shenzhen, Guangdong People’s Republic of China; 3 Department of Orthopaedics, General Hospital of Central Theater Command, Wuhan, Hubei People’s Republic of China; 4 Department of Orthopaedics, South China Hospital of Shenzhen University, No. 1 Fuxin Road, Longgang District, Shenzhen, 518111 Guangdong People’s Republic of China

**Keywords:** Anatomy, Neurology, Risk factors, Signs and symptoms

## Abstract

This study aims to investigate the feasibility and efficacy of anterior atlantoaxial motion preservation fixation (AMPF) in treating axis complex fractures involving the odontoid process fracture and Hangman’s fractures with C2/3 instability. A retrospective study was conducted on eight patients who underwent AMPF for axis complex fractures at the General Hospital of Central Theater Command from February 2004 to October 2021. The types of axis injuries, reasons for injuries, surgery time, intraoperative blood loss, spinal cord injury classification (American Spinal Injury Association, ASIA), as well as complications and technical notes, were documented. This study included eight cases of type II Hangman’s fracture, five cases of type II and three cases of type III odontoid process fracture. Five patients experienced traffic accidents, while three patients experienced falling injuries. All patients underwent AMPF surgery with an average intraoperative blood loss of 288.75 mL and a duration of 174.5 min. Two patients experienced dysphagia 1 month after surgery. The patients were followed up for an average of 15.63 months. One case improved from C to E in terms of neurological condition, three cases improved from D to E, and four cases remained at E. Bony fusion and Atlantoaxial Motion Preservation were successfully achieved for all eight patients. AMPF is a feasible and effective way for simultaneous odontoid process fracture and Hangman’s fractures with C2/3 instability, while preserving atlantoaxial movement.

## Introduction

Axis traumatic fractures are common injuries in the cervical region^1^. Conservative treatment using a cervical collar or rigid external fixation has shown satisfactory results for stable fracture types^2^. However, unstable fractures associated with neurological injury often require surgical intervention. Previous surgical studies have primarily focused on single Hangman’s fracture or odontoid fracture^3,4^. Nevertheless, it is not uncommon to encounter axis complex injuries involving simultaneous odontoid process fracture and Hangman’s fracture^5,6^. The optimal surgical approach for these axis complex fractures remains controversial. This retrospective study aims to preliminarily evaluate the feasibility and efficacy of anterior Atlantoaxial Motion Preservation fixation (AMPF), which involves an anterior approach single odontoid process screw and C2/C3 discectomy with intervertebral fusion using plate and screw fixation, for the treatment of unstable axis complex fractures.

## Methods

### Patients

A retrospective analysis was conducted using the medical records of 65 patients with axis fractures treated at the General Hospital of Central Theater Command from February 2004 to October 2021. The inclusion criteria for this study were as follows: (1) Axis complex fractures involving simultaneous fractures of the axis arch and odontoid process. (2) Underwent anterior Atlantoaxial Motion Preservation fixation (AMPF) surgery. (3) Followed up for a minimum of 1 year after cervical AMPF surgery. (4) Provided consent to participate in this retrospective study and the publication of research findings. Exclusion criteria included: (1) Rupture of the transverse ligament of the atlas. (2) Axis burst, comminuted fractures, or irreducible conditions. (3) Presence of pathological or congenital odontoid process lesions. (4) Axis complex fracture patients who did not undergo cervical surgery due to severe injuries of other organs. (5) Injuries associated with atlantooccipital instability or C3 fractures requiring posterior fixation. A total of eight patients’ data met the inclusion and exclusion criteria and were included in the study. Prior to surgery, all patients provided informed consent. The surgeries were performed by a single senior surgeon (Prof. CAI). This retrospective study was approved by the Research Ethics Committee of Wuhan Medical Service and the Institutional Review Boards of the General Hospital of Central Theater Command (No. 0090611-3). All study procedures were performed following the relevant guidelines and principles of the Declaration of Helsinki. Signed informed consents were obtained from all participants to participate in this study.

### Preoperative management

Prior to surgery, the following steps were taken to ensure appropriate patient management: (1) Prioritization of patients: Patients with intracranial, abdominal, and open injuries were prioritized to stabilize their vital signs. (2) Diagnostic imaging: Preoperative X-rays (including anteroposterior, lateral, and open-mouth views) and CT scans were conducted to assess the condition of the odontoid process, axis vertebra body and arch. MRI scans were performed to evaluate soft tissue elements such as the spinal cord, ligaments, discs, and hematoma. (3) Assessment of neurological deficits: For patients with neurological deficits, the spinal cord injury condition was documented using the ASIA classification. Skull traction, with a weight of 3–5 kg, was employed to achieve reduction and reduce spinal cord irritation once patients were admitted to the hospital. (4) Patient education and consent: Detailed information regarding the injury condition, surgical procedure, and associated risks were provided to patients and their families. Informed consent was obtained from patients and their families after thorough explanation. By implementing these preoperative measures, doctors ensured a comprehensive assessment of the patients’ condition and obtained informed consent, thereby promoting patient safety and effective surgical management.

### Surgical procedure

**Figure 1:**
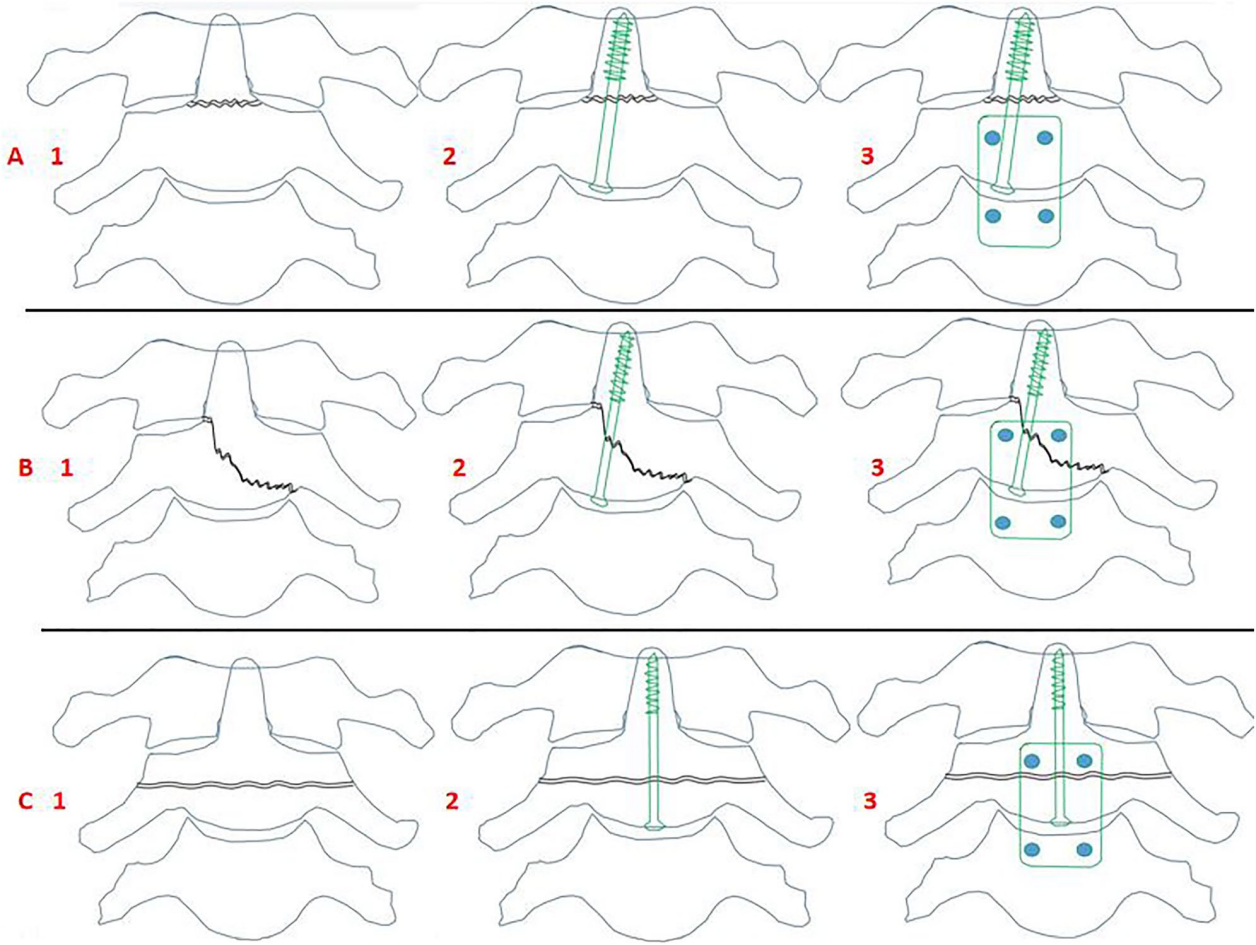
Different fracture patterns and fixation ways: (**A1**) Type II odontoid fracture. (**A2**) Slight oblique placement of odontoid process screw. (**B1**) Type III odontoid fracture with oblique fracture line. (**B2**) Slight oblique placement of odontoid process screw. (**C1**) Type III odontoid fracture with transverse fracture line. (**C2**) Neutral placement of odontoid process screw. (**A3,B3,C3**) Anterior plate and screws fixation.

**Figure 2:**
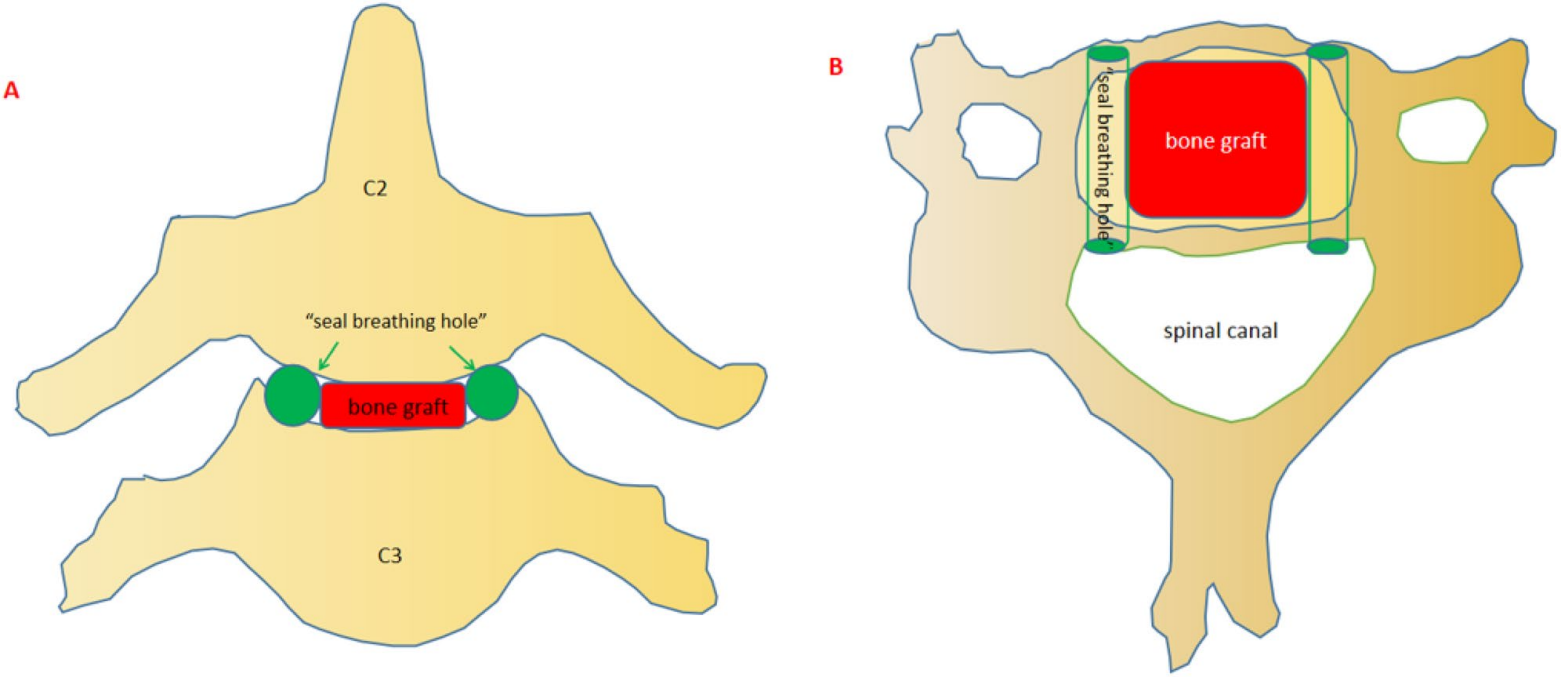
Bilateral or unilateral tunnels (green parts in **A,B**) were created beside intervertebral autogenous iliac bone (red part in **A,B**) to prevent postoperative intraspinal hematoma.

The patient was placed in the supine position under general anesthesia, with the head and cervical region adjusted to an extended position using skull traction. Continuous neuroelectrophysiological monitoring was maintained throughout the surgery. A transverse incision was made in the submaxillary skin on the right side of the neck. The subcutaneous fat and platysma myoides were dissected horizontally to access the cervical column by creating a surgical corridor between the cervical vessel sheath and the thyrohyoid muscle. To enhance visualization, a S-shaped retractor was employed to lift the submandibular gland upward. The C2/3 discectomy was carried out from the upper side of the superior thyroid artery to expose the spinal canal. Any small bone fragments and hematoma present in the spinal canal were meticulously removed with loops and light-assisted for chief surgeon. Satisfactory reduction of the fracture was achieved under skull traction, with its accuracy confirmed using fluoroscopy. Depending on the type of odontoid fracture, the odontoid process screw guide pin was inserted as follows: (1) For type II odontoid fracture (Anderson-D’Alonzo classification) (Fig. 1A1,B1) and type III with oblique fracture line involving the bottom of odontoid process base, the odontoid process screw guide pin was placed slightly obliquely (Fig. 1A2,B2). (2) For type III with a transverse fracture line on the axis vertebral body (Fig. 1C1), the guide pin was neutrally placed on the odontoid process (Fig. 1C2). After fluoroscopy confirmed the guide pin’s position and length, a screw track was prepared along the guide pin. A cannulated lag screw with the appropriate length was advanced along the pin. Autogenous iliac bone grafting was harvested and placed in C2/3 with a “seal breathing hole” (Fig. 2A,B) in the space. An anterior cervical plate was positioned either in or not in the middle line of the axis vertebral body and secured by four screws according to the fracture line (Fig. 1A3,B3,C3). Closure of the wound was performed in layers with a drainage tube included.

### Postoperative management

After the surgery, the following postoperative management protocols were implemented: (1) The drainage tube was removed 48 h after the surgery to facilitate wound healing. (2) All patients received prophylactic antibiotics to prevent infection. Sutures on the incision were removed after 8 days, ensuring proper wound healing. (3) Cervical collar: Patients were instructed to wear a cervical collar for 6 weeks post-surgery to provide support and stability to the cervical region. (4) All patients were followed up for a minimum of 12 months. During each follow-up visit, clinical evaluations were conducted using the ASIA classification system to assess neurological function. Additionally, X-rays and CT scans were performed to monitor the progress of healing and the stability of the fixation.

## Results

**Figure 3:**
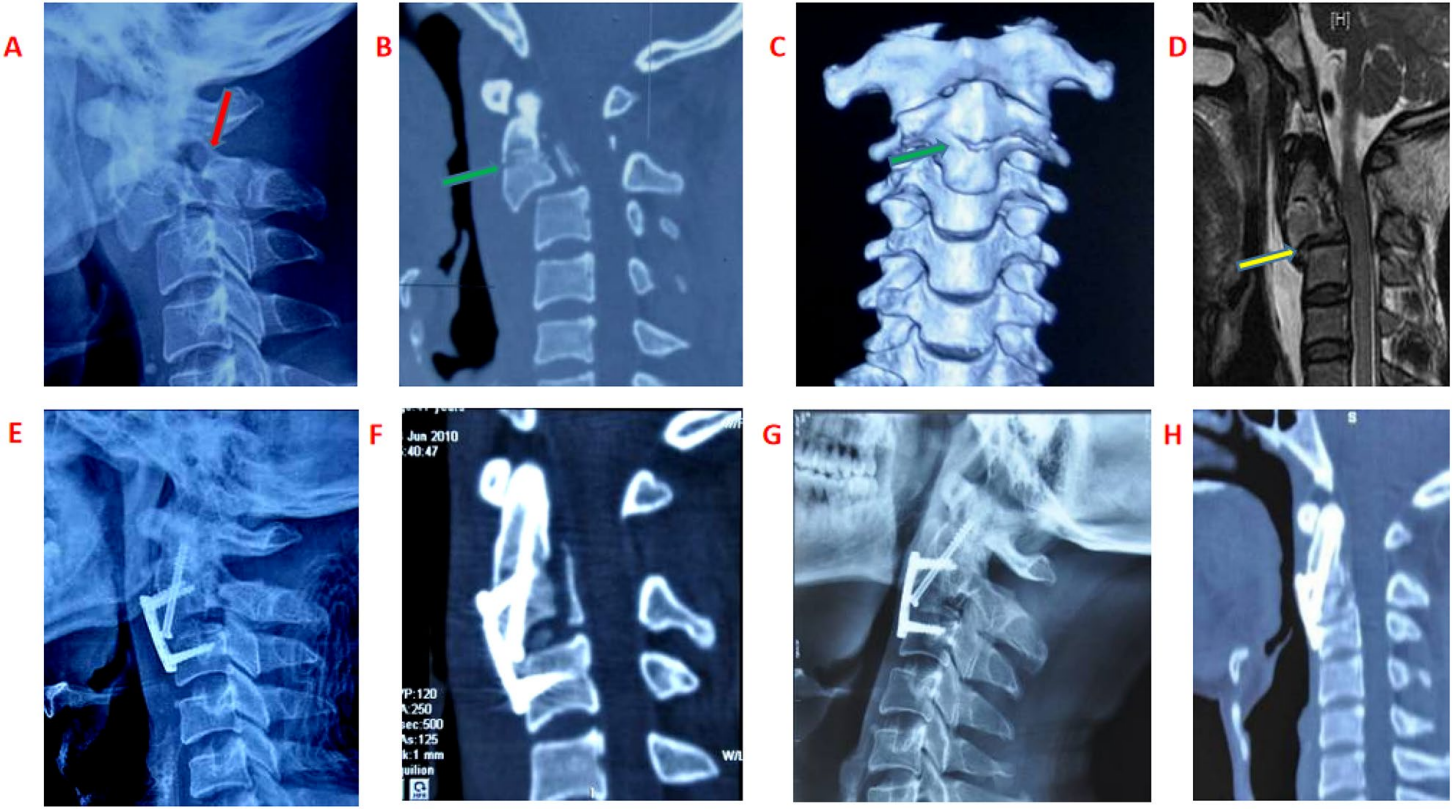
Typical case: 42 years male patient was admitted for traffic accident injury. (**A**) Pars interarticularis of the neural arch fractures (red arrow). (**B**) Odontoid process fracture line in AP view (green arrow). (**C**) Odontoid process fracture with transverse fracture line on CT (green arrow). (**D**) C2/3disc rupture (yellow arrow). (**E**) AP view of X ray after AMPF (8 days). (**F**) CT scanning after AMPF (8 days). (**G**) AP view of X ray after AMPF (12 months) with stable fixation. (**H**) CT scanning after AMPF (12 months) with satisfied osteonal union.

A total of eight patients, including seven males and one female, with an age range of 27 to 53 years old, underwent AMPF surgery and were followed up for a period of 13 to 24 months. The neurological outcomes of all patients were assessed and showed satisfactory results 12 months after surgery. The detailed information regarding the perioperative period and follow-up is presented in Table 1. Significantly, all eight patients successfully preserved atlantoaxial movement following the surgical procedure. However, two patients experienced dysphagia symptoms one month after surgery. Fortunately, these symptoms gradually reduced with the implementation of physical therapy and oral medication. These results highlight the positive outcomes achieved through the utilization of AMPF surgery in the treatment of axis complex fractures, specifically odontoid process fractures combined with hangman’s fractures and C2/3 instability. A typical case was presented, illustrating the successful outcome, as shown in Fig. 3A–H.

**Table 1:** Specific informations about patients in perioperative and follow up period.

Case	Gender	Age (years)	Hangman’s fracture type	Odontoid process fracture type	Associated with other injuries	Injury reason	Surgery duration for axis injury (min)	Blood loss (ml)	Bone heal and atlantoaxial motion perservation	Pre/post (12 months later) ASIA
1	M	34	II	II	Spleen rupture, hemorrhagic shock, C2/3 disc rupture	Traffic accident	230	750	Yes	D/E
2	M	47	II	III with transverse fracture line	Brain injury (mild), right clavicle fracture, C2/3 disc rupture	Falling	150	230	Yes	E/E
3	M	42	II	III with transverse fracture line	C2 body posterior margin fracture, brain injury (mild), C2/3 disc rupture	Traffic accident	149	245	Yes	E/E
4	M	41	II	II	Right brachial plexus injury, right clavicle fracture, C2/3 disc rupture	Traffic accident	185	320	Yes	D/E
5	M	45	II	II	Left 6th ribs, left tibia and fibula fracture, C2/3 disc rupture	Falling	190	350	Yes	E/E
6	M	48	II	III with oblique fracture line	Axis body anterior tear fracture, C2/3 disc rupture	Traffic accident	182	170	Yes	D/E
7	M	53	II	II	Mandible fracture, brain injury (mild), C2/3 disc rupture	Falling	175	150	yes	C/E
8	F	27	II	II	Fracture of right radius and ulna, C2/3 disc rupture	Traffic accident	135	95	Yes	E/E

## Discussion

### Epidemiological condition of axis complex fractures

Axis fractures constitute approximately 20% of all acute cervical fractures^7^. Complex fractures of the axis, often associated with odontoid and Hangman’s fractures, are not uncommon, primarily due to high-energy trauma^5^. Hangman’s fracture, also termed traumatic spondylolisthesis of the axis, was initially described by Schneider in 1965 and is characterized by fractures to the neural arch, articular facets, or pars of the axis vertebra^8^. Younger patients commonly experience odontoid fractures resulting from high-energy trauma, such as motor vehicle accidents, while older patients are more susceptible to fractures from falls. Odontoid fractures typically occur at the tip, through the waist, or on the vertebral body^9^. Axis complex fractures encompass multiple lesions primarily involving the neural arch, odontoid process, or vertebral body. Despite the individual study focus on Hangman’s fractures and odontoid fractures for several decades, research on axis complex fractures remains relatively limited. In our study, based on our statistical analysis, we observed that axis complex fractures (8 cases) accounted for nearly one-eighth of all axis fracture patients (65 cases) during our consecutive observation period. This prevalence aligns with findings reported in a study by Korres DS^5^.

### Analysis of axis complex fractures mechanism

Simultaneous fractures of the odontoid process and neural arch are the most frequently observed pattern in axis complex fractures^10^. This suggests that these fractures may be caused by the same forces acting simultaneously, indicating a potential common injury mechanism between odontoid and Hangman’s fractures. Initially considered a hyperextension distraction injury, Hangman’s fracture was first described in a victim of judicial hanging; however, subsequent studies proposed mechanisms involving extension-compression and flexion-compression^11,12^. The classification of Hangman’s fracture, initially proposed by Effffendi et al. and later modified by Levine and Edwards, is widely used to evaluate the degree of injury and guide treatment decisions^13,14^. Type II Hangman’s fracture is associated with upper cervical extension and axial compression load. In the case of odontoid fractures, the Anderson-D’Alonzo classification is universally used by scholars^15^. Odontoid fractures represent the most prevalent pattern observed in axis fractures. Biomechanical studies indicate that forces acting on the upper forehead result in rapid deceleration of the head combined with continued momentum of the torso, leading to extension-compression and anterior shear forces, particularly in odontoid process type II and III fractures^16^. In our study, all eight cases were associated with type II Hangman’s fractures, suggesting a likelihood of odontoid fractures merging with extension-compression forces to induce type II Hangman’s fractures. Type IIa and type III Hangman’s fractures are primarily induced by flexion-distraction and flexion-compression forces, which may not align well with the injury mechanism of odontoid fractures^12^. These complex axis fractures are often accompanied by C2/3 disc rupture. The odontoid process may break first under an extension-compression combination with anterior shear force, and this force may then be transmitted to the neural arches, causing damage. Conversely, if the neural arches of the axis fracture first, the posterior support force would be lost, and the anterior shear force would decrease significantly, potentially failing to subsequently fracture the odontoid process. Therefore, we speculate that odontoid process injury may occur prior to axis neural arch fractures in this pattern of complex fractures.

### Previous treatment methods for axis complex fractures

Nonoperative treatment involving traction and external immobilization, such as halo vest orthoses and cervical collar, can yield satisfactory results for the majority of stable fractures of the odontoid or neural arch^17,18^. In the case of unstable odontoid fracture, anterior odontoid process screw fixation is a widely accepted approach, with studies demonstrating the adequacy of a single screw fixation^19–22^. Other surgical techniques, such as C1–C2 fusion with sublaminar wire fixation and bone grafting (Brooks technique, Gallie technique), C1–C2 fusion with transarticular screw fixation (Magerl and Seemann technique), and C1–C2 fusion with C1 lateral mass and C2 pedicle screw-rod fixation (Harms technique), can also be used to treat odontoid fractures. However, it’s important to note that these techniques may result in the loss of atlantoaxial motion^9^. Surgical intervention is recommended for unstable Hangman’s fractures, particularly Levine–Edwards Type II, IIa, and III fractures with significant dislocation^18^. Surgical approaches for Hangman’s fractures include anterior, posterior, and anterior–posterior combination approaches. Anterior C2/C3 discectomy with fusion has been advocated due to its advantages of shorter operative duration and lower blood loss^12,23^. In the case of axis complex fractures involving both the odontoid process and Hangman’s fracture, surgical intervention is a reasonable option, given the instability of the injury. However, there is no standardized method available for these complex fractures^5^. While posterior approaches with fixation of C1, C2, and/or C3 can restore and stabilize the cervical segment, they often sacrifice atlantoaxial movement function^24–26^. Zhu^27^ introduced anterior odontoid process screw fixation and posterior percutaneous screw fixation using intraoperative O-arm navigation as a method to preserve atlantoaxial movement in the treatment of axis injuries involving type III odontoid process fractures and type I Hangman’s fractures. However, this method requires both anterior and posterior approaches and expensive surgical equipment.

### Atlantoaxial motion preservation fixation procedure for axis complex fractures

We proposed this atlantoaxial motion preservation fixation (AMPF) method and performed on October 10th, 2005, for our first case of odontoid and Hangman’s fracture using this technique. A total of eight consecutive cases were documented using this method. During the surgical procedure, intraoperative fluoroscopy was used to achieve reduction of the odontoid process before placing a single odontoid process lag screw following the guide pin. For type II odontoid fractures and type III fractures with an oblique fracture line involving the bottom of the odontoid process base, we suggested placing the lag screw slightly obliquely to enhance anti-rotation ability and improve fusion. Following the odontoid process fixation, C2/3 discectomy was performed. Manual adjustment was performed, if necessary, to achieve reduction of the neural arch under fluoroscopy guidance. One potential complication after cervical fusion surgery is intraspinal hemorrhage. We observed that traumatic fractures of the cervical spine tend to result in more bleeding during and after surgery compared to surgeries for degenerative cervical disorders. To mitigate the risk of intraspinal hemorrhage during the drainage period after surgery, we left unilateral or bilateral channels beside the autogenous iliac bone in the C2/3 intervertebral space during the surgical procedure. Complete removal of the C2/3 disc tissue and medial luschaka joint resection were performed. The width of the autogenous iliac bone graft was appropriately controlled, leaving one or two channels instead of fully filling the intervertebral space in width. The creation of passageways (seal breathing hole) in the intervertebral space was undertaken to reduce the risk of spinal cord compression from intraspinal hemorrhage during the drainage period after surgery. This concept is similar to the principle of providing a breathing channel through a hole in thick ice, establishing a pathway for seal respiration in an enclosed environment (Fig. 2). The placement of an anterior cervical plate was determined based on the fracture line of the axis vertebral body. It was fixed in the middle line using four screws, providing enhanced immobilization properties to promote bone healing. Our previous biomechanical study conducted in 2014 demonstrated the satisfactory biomechanical properties of the AMPF technique in treating multiple axis injuries^28^. Another biomechanical study by Hu Y confirmed the excellent biomechanical performance of anterior odontoid process screw plate with bone graft in the treatment of type II odontoid process and type I hangman fractures with C2–3 disc rupture^29^. Additionally, Heiko Koller introduced the “Simultaneous anterior arthrodesis C2–3 and anterior odontoid screw fixation” technique in 2006, and Benjamin Blondel introduced the “single anterior procedure for stabilization” technique in 2009, both of which aimed to treat this type of axis injury in one case report and achieved reliable stability and good function^30,31^. Inevitablely, residual deformity after surgery in C2/3 was not rare. Residual deformity after ACDF might cause potential problems over the long-term^32,33^. In our study of eight cases, the preservation of rotational movement of the atlanto-axial articulation was achieved using the AMPF method. This technique effectively managed odontoid and Hangman’s fractures, restoring stability to the cervical spine. Additionally, it was observed that the AMPF procedure from an anterior approach could also address tear drop fractures from the posterior margin and intraspinal hematoma. The neurological injuries in the cases included in this study were incomplete, and the clinical symptoms were mild. The neurological outcomes were satisfactory, indicating that favorable prognoses can be achieved with timely decompression and the restoration of stability.

### Technique notes regarding the AMPF method for treating axis complex fractures with C2/3 instability

(1) *Imaging examination* CT scanning is accurate for evaluating bone lesions in the odontoid process, vertebral body, and posterior arch. MRI is more sensitive in assessing soft tissues such as the transverse ligament of atlas and intervertebral disc (C2/3). X-ray can assist in fracture classification. (2) *Fracture line and ligament stability* Fracture lines that extend from high to low anterior of the odontoid process are not suitable for the AMPF method, as the upper odontoid process trend to forward dislocated as the cannulated lag screw began to compress. Additionally, rupture of the transverse ligament of atlas leads to atlantoaxial joint instability, making it unsuitable for AMPF. (3) *Spinal cord injury and reduction* Maintaining a balance between spinal cord injury and ideal reduction is crucial. Mediated skull traction is performed when the patient is in a clear state of consciousness, and bedside fluoroscopy is used to check the traction weight, ensuring satisfactory reduction in the ward. In the operating room, the patient’s surgical position is carefully set with ideal traction weight and a clear state of consciousness before undergoing anesthesia. (4) *Anesthesia and monitoring* General anesthesia through a nasal tube, rather than mouth, and a cervical spine extension position facilitate exposure and subsequent operative procedures. Neuroelectrophysiological monitoring is essential during surgery. (5) *Guide pin insertion and lag screw length* Accurate insertion of the guide pin and an appropriate length of the cannulated lag screw are crucial to avoid complications related to bone penetration and provide sufficient fixation force. (6) *Management of young patient’s disc* Unlike degenerated cervical intervertebral discs, the discs of young patients are not easily managed with conventional curettes. The use of a high-speed burr is an effective instrument in such cases. (7) *Controlled screw advancement* Excessive pressure during the advancement of the cannulated lag screw should be avoided to prevent over-cutting of the bone and reduce holding force, which could lead to fixation failure.

### Limitations

This study did not encompass axis complex injuries of type I, IIa, and type III Hangman’s fracture. Further exploration, with larger sample sizes and biomechanical studies, is necessary to analyze the reasons behind this limitation. Elderly patients with osteoporosis were not included in this study, and the effectiveness of the AMPF method in this population requires further investigation.

### Conclusion

The AMPF method show both feasibility and satisfactory efficacy in treating axis complex fractures that involve simultaneous odontoid process fracture, Hangman’s fracture, and C2/3 instability while preserving atlantoaxial movement. In comparison to posterior approach methods, AMPF presents advantages in managing the anterior longitudinal ligament, cervical disc, tear drop fractures of the posterior margin of the axis body, and intraspinal hematoma. Consequently, AMPF emerges as a viable alternative for addressing this type of axis complex injury.

### Ethics declarations

Informed and publish report consents were got from all patients. This retrospective study was approved by the Research Ethics Committee of Wuhan Medical Service and Institutional Review Boards of the General Hospital of Central Theater Command (No. 0090611-3).

## Data Availability

The datasets generated and/or analyzed during the current study are available from the corresponding author on reasonable request.
